# Correction: Dual luminescence and infrared circularly polarized luminescence up to 900 nm with platinum complexes bearing a helical donor–acceptor ligand

**DOI:** 10.1039/d6qm90042a

**Published:** 2026-06-29

**Authors:** Pablo Vázquez-Domínguez, Maher Hojorat, Eva Suits, Francisco José Fernández de Córdova, Nicolas Vanthuyne, Denis Jacquemin, Abel Ros, Ludovic Favereau

**Affiliations:** a Institute for Chemical Research (CSIC-US) C/Américo Vespucio 49 E-41092 Seville Spain abel.ros@iiq.csic.es; b Department of Organic Chemistry, Innovation Centre in Advanced Chemistry, ORFEO-CINQA, University of Seville C/Prof. García González 1 41012 Seville Spain; c Univ Rennes, CNRS, ISCR-UMR 6226 F-35000 Rennes France ludovic.favereau@univ-rennes.fr; d Aix Marseille University, CNRS, Centrale Marseille, iSm2 Marseille France; e Nantes Université, CNRS, CEISAM UMR 6230 F-44000 Nantes France Denis.Jacquemin@univ-nantes.fr; f Institut Universitaire de France (IUF) F-75005, Paris France

## Abstract

Correction for ‘Dual luminescence and infrared circularly polarized luminescence up to 900 nm with platinum complexes bearing a helical donor–acceptor ligand’ by Pablo Vázquez-Domínguez *et al.*, *Mater. Chem. Front.*, 2024, **8**, 3799–3806, https://doi.org/10.1039/D4QM00632A.

The authors regret that the name of one of the authors (Maher Hojorat) was shown incorrectly in the original article. The corrected author list is as shown above.

The Royal Society of Chemistry apologises for these errors and any consequent inconvenience to authors and readers.

